# Case Report: Retroperitoneal Sarcoma in Six Operations: Our Experience in Operative Management of Blood Vessels

**DOI:** 10.3389/fonc.2022.885033

**Published:** 2022-04-28

**Authors:** Jinhui Guo, Fabo Qiu, Jie Zhao, Qiliang Lu, Wen Fu, Qiuran Xu, Dongsheng Huang

**Affiliations:** ^1^Qingdao Medical College, Qingdao University, Qingdao, China; ^2^Laboratory of Tumor Molecular Diagnosis and Individualized Medicine of Zhejiang Province, Zhejiang Provincial People’s Hospital, Affiliated People’s Hospital, Hangzhou Medical College, Hangzhou, China; ^3^Department of Hepatopancreatobiliary Surgery, The Affiliated Hospital of Qingdao University, Qingdao, China; ^4^College of Biotechnology and Bioengineering, Zhejiang University of Technology, Hangzhou, China

**Keywords:** retroperitoneal dedifferentiated liposarcoma, myxofibrosarcoma, rhabdomyosarcoma differentiation, recurrent, vascular resection

## Abstract

Here we introduce a case of retroperitoneal liposarcoma, which is characterized by repeated recurrences after surgery, and has undergone a total of 6 operations. The diameter of the tumor was about 26 cm at the time of the patient's diagnosis. The imaging examination revealed that the surrounding organs and blood vessels were invaded, which brought great challenges to radical resection. The postoperative pathology of the patient’s first operation was dedifferentiated liposarcoma, and some areas showed myxofibrosarcoma differentiation. With the recurrence of sarcoma, myxofibrosarcoma dedifferentiated into rhabdomyosarcoma, and malignant fibrous histiocytoma appeared in some areas. How to treat this type of patient after recurrence? How to deal with blood vessels wrapped by sarcoma during surgery? The medical community has not yet reached the same conclusion. We describe the process of treating the patient and the experience of dealing with blood vessels during surgery.

## Introduction

Retroperitoneal liposarcoma is a malignant tumor that originates in soft tissues. Compared with other types of tumors, its biggest feature is a significant local recurrence rate (1–3). Low-grade liposarcoma may not change for a long time in the patient's body, but once the sarcoma has poorly differentiated components, the sarcoma will progress rapidly and the waist circumference will also increase. Due to the limited effect of adjuvant therapy, surgical resection is still the only way to cure the disease in patients with retroperitoneal liposarcoma (4, 5). A persistent problem after the first surgical resection is repeated recurrence of sarcoma. Up to 69-80% of first recurrence cases will have multiple local recurrences (6, 7).

## History of Surgery

### 2012-03-14

**Figure 1:**
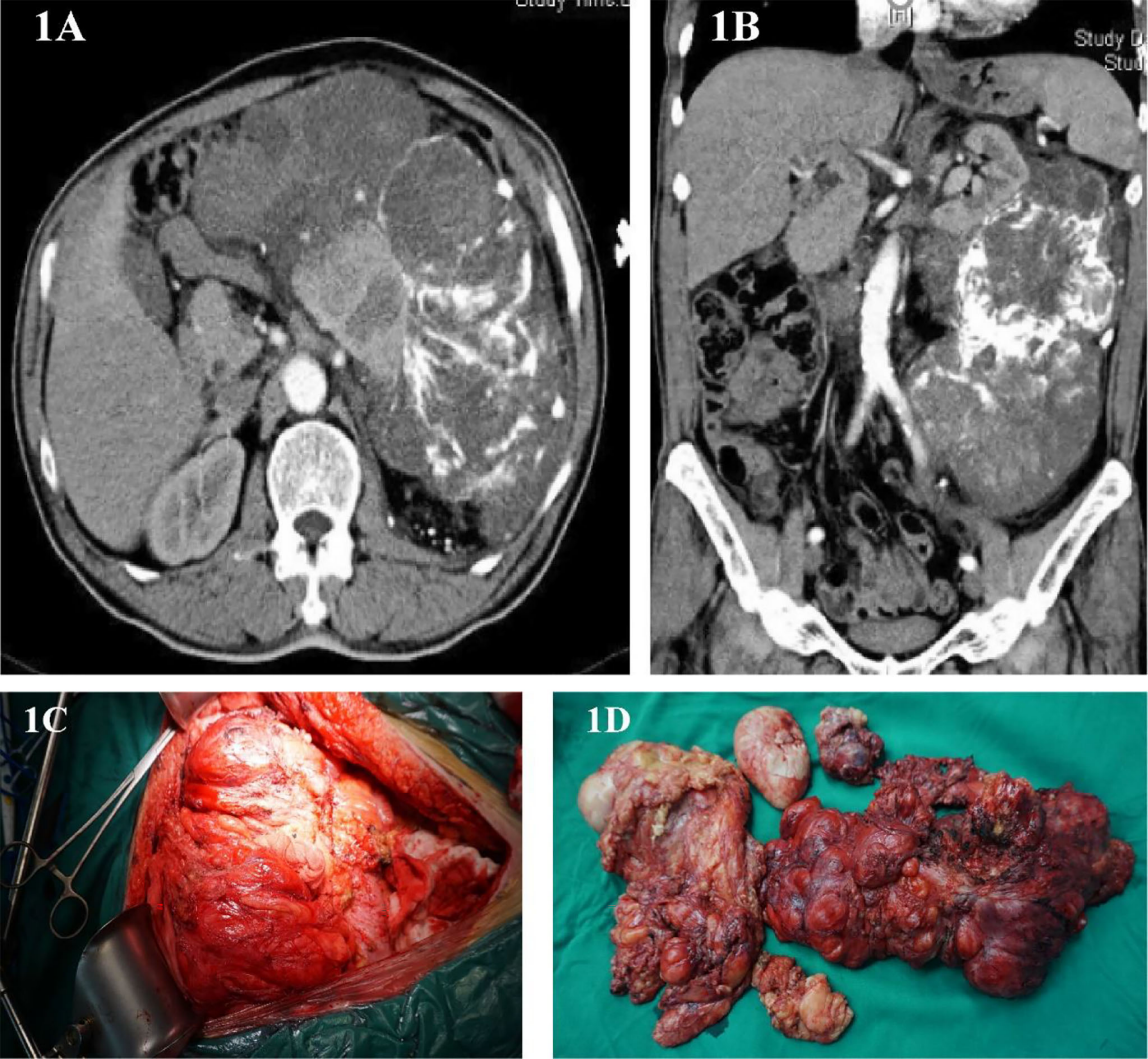
**(A, B)** Abdominal image before the first operation. **(C)** Intraoperative photos. **(D)** Surgical piece of the dedifferentiated liposarcoma.

This is a 60-year-old male who found an abdominal mass for 8 months. His waist circumference gradually increased, during which no treatment was taken. The patient was in good health and had no history of cancer. In March 2012, he was hospitalized due to upper abdomen discomfort. Physical examination of his upper abdomen revealed a huge mass. Tumor markers such as carcinoembryonic antigen (CEA), carbohydrate antigen199 (CA-199), carbohydrate antigen 125 (CA125), carbohydrate antigen 50(CA50), alpha-fetoprotein (AFP) are all within the normal range. Abdominal CT scans showed a huge lump around the left kidney, considering malignant tumors (**Figures 1A, B**). On March 14, 2012, we performed a radical surgical resection on the patient. Operation name: retroperitoneal tumor resection+ left nephrectomy+ left adrenalectomy. Part of the tumor is tough, most of it is hard and bony, which surrounds the kidney and adheres to the surface of the kidney. During the operation, the kidney could not be stripped, and it is suspected that the sarcoma has invaded the kidney (**Figure 1C**). In order to maximize the marginal negative resection, so as to reduce the recurrence rate of tumor after surgery. The surgeon decided to perform combined organ resection and removed the left kidney and left adrenal gland (**Figure 1D**). The postoperative pathology was dedifferentiated liposarcoma, and the dedifferentiated component was like myxofibrosarcoma, which invaded the kidney tissue, and did not involve the ureteral stump and adrenal gland. Tumor components were seen around the renal hilar vessels. The patient’s vital signs were stable after surgery and was discharged from hospital on March 22, 2012.

### 2013-02-22

**Figure 2:**
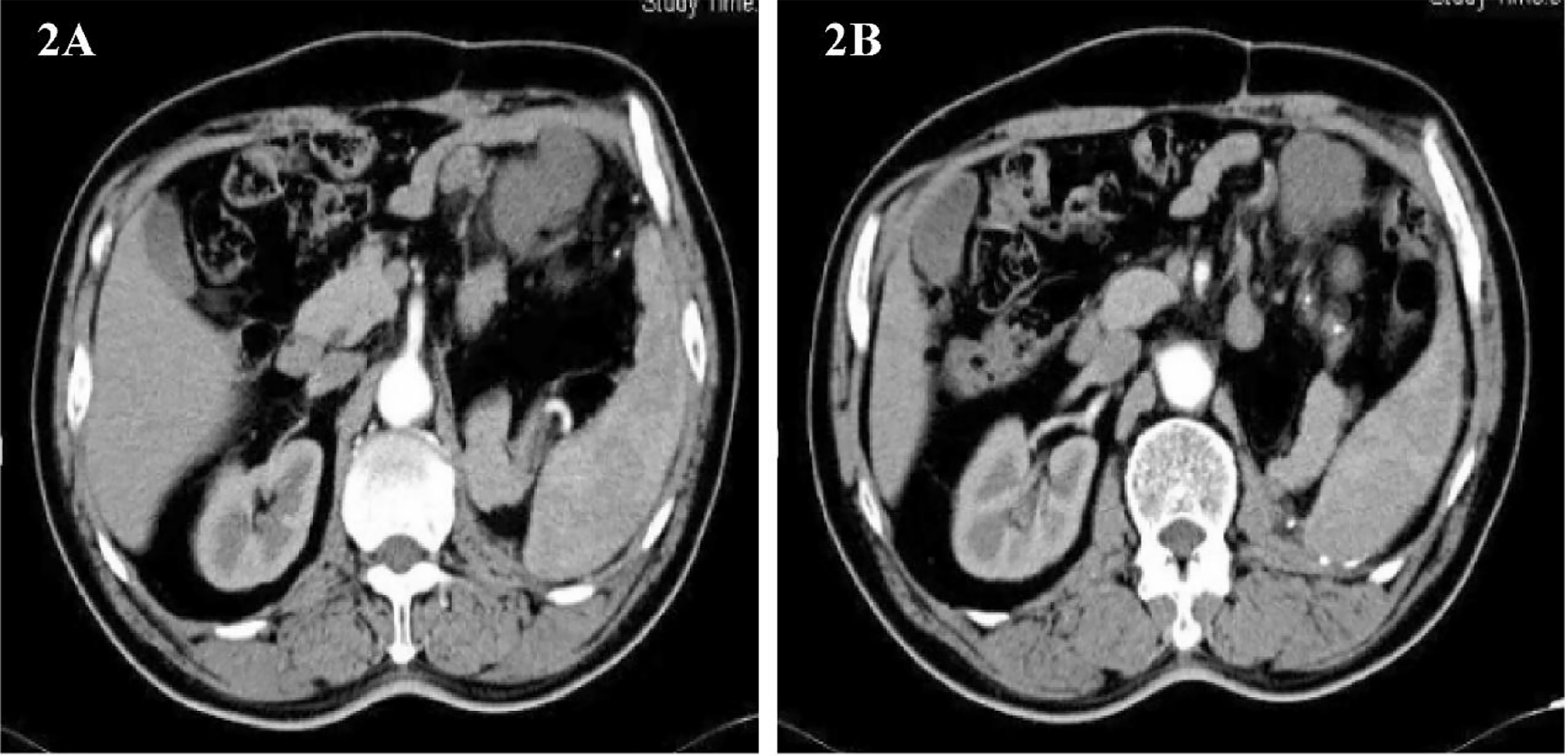
**(A, B)** Abdominal image before the second operation.

Imaging follow-up found that the tumor recurred, and it has been less than 1 year since the patient’s last surgery. Abdominal CT showed a round, slightly low-density foci in the upper left abdominal cavity with enlarged adjacent lymph nodes(**Figures 2A, B**). The patient was soon admitted to the hospital for surgery for the second time. On February 22, 2013, the same surgical team performed a combined organ resection for the patient. Operation name: Abdominal tumor resection + partial resection of small intestine. The postoperative pathology was dedifferentiated liposarcoma, and the dedifferentiated components were like myxofibrosarcoma. Immunohistochemistry : CD34(+), Caldesmon(+), SMA(+), CD117(-), Dog-1(-), S-100(-). The Ki67 positive rate is about 30%. The patient’s gastrointestinal motility recovered on the third postoperative day, and the diet gradually transitioned from a liquid diet to a normal diet. The patient was discharged from the hospital on March 1, 2013.

### 2014-06-09

**Figure 3:**
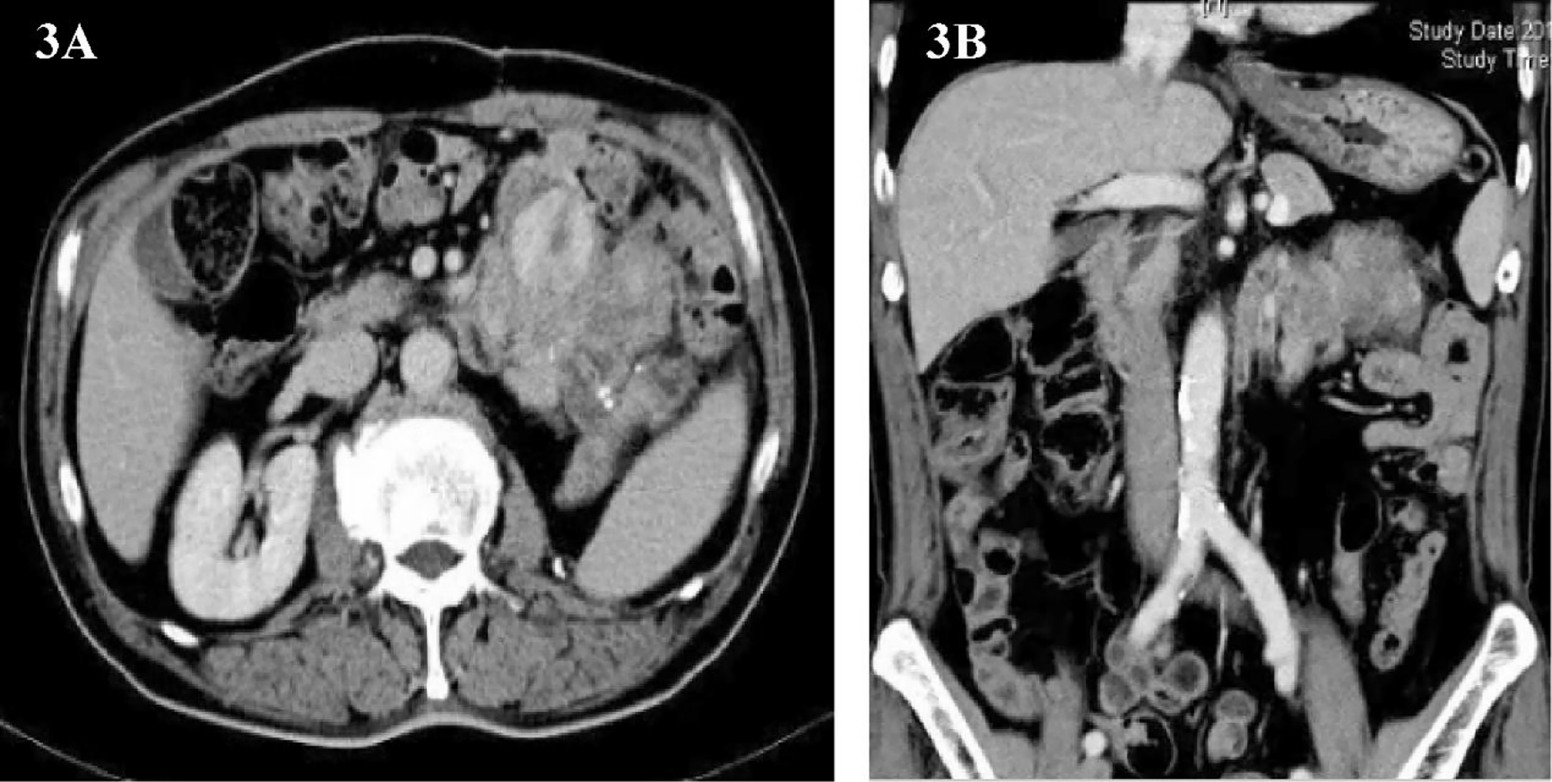
**(A, B)** Abdominal image before the third operation.

One and a half years later, the patient’s imaging examination revealed that the tumor had recurred again (**Figures 3A, B**). We performed the third operation for the patient on June 9, 2014. Operation name: Resection of retroperitoneal tumor + partial resection of colon splenic flexure. The postoperative pathology was dedifferentiated liposarcoma, and some areas showed rhabdomyosarcoma differentiation. Immunohistochemistry : MyoD1(+), Myogenin (+), SMA(+), CD117(+), Caldesmon(+), CD34(-), Dog-1(-), S-100(-). The Ki67 positive rate is about 30%. The postoperative nutritional status of the patient was poor, and the abdominal incision appeared fat liquefaction. We change the dressing for patients twice a day. The patient’s abdominal wall incision was dehisced on the 8th postoperative day, and he entered the operating room again for debridement and suture. After the operation, the patient was given anti-infection and nutritional support, and the stitches were removed smoothly two weeks later. The patient was discharged on July 15, 2014.

### 2015-01-21

**Figure 4:**
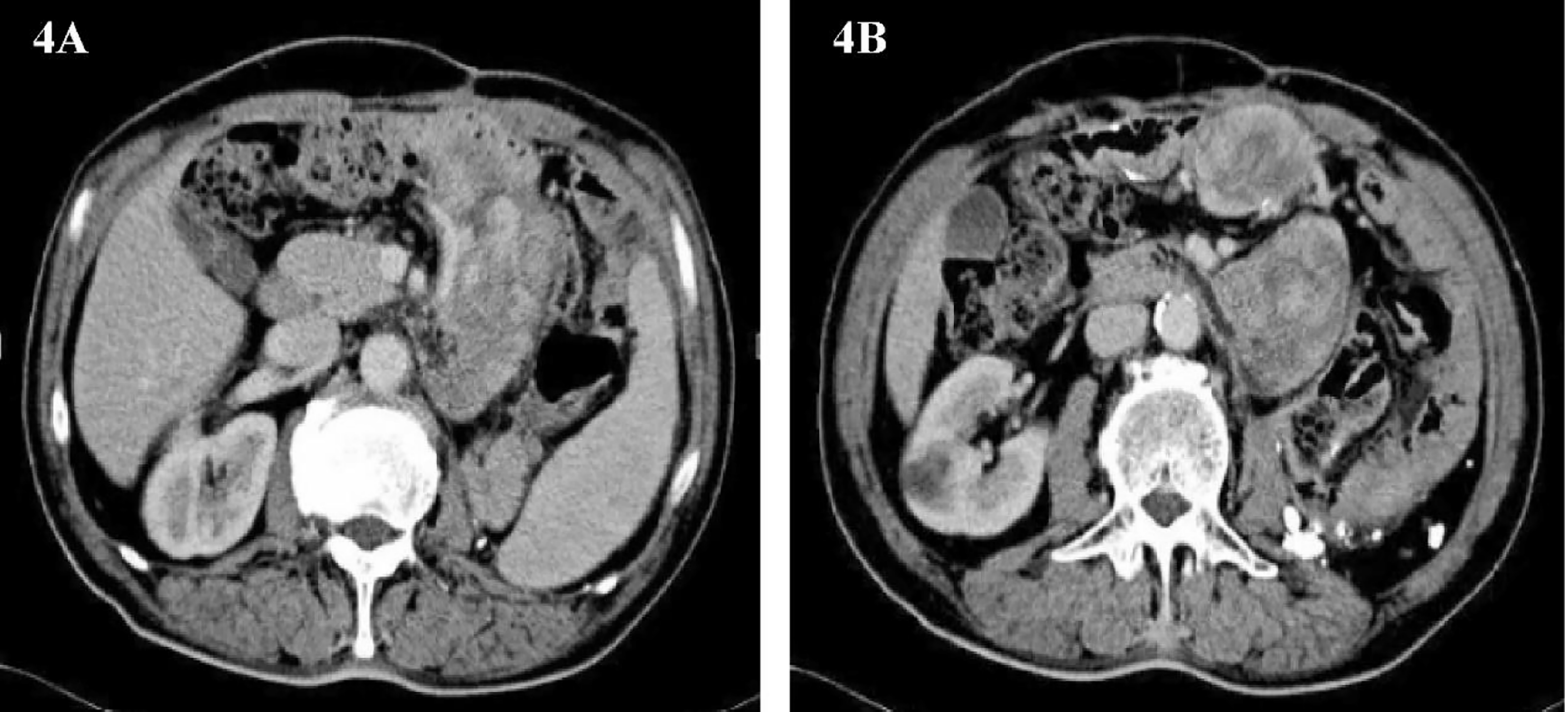
**(A, B)** Abdominal image before the fourth operation.

Only 3 months have passed since the last operation, the imaging examination showed that the tumor had recurred. In consideration of the patient’s physical condition, no treatment will be carried out temporarily, and the patient will be asked to recheck regularly. After 3 months, abdominal CT showed multiple masses of soft tissue density shadow in the abdominal cavity, and the lesions were significantly larger than before (**Figures 4A, B****)**. Only half a year after the patient’s last operation, we decided to have a multidisciplinary consultation. The chief surgeon invited experts from oncology, pathology, radiotherapy, medical imaging and urology departments to discuss. After multi-disciplinary discussion, it is recommended to perform surgery for the patient again. On January 21, 2015, we performed the fourth operation for the patient. Operation name: Resection of multiple retroperitoneum tumors + Partial jejunum resection + Partial resection of the ileum + Duodenal jejunum end-to-end anastomosis + Partial resection of small mesentery + Partial splenectomy + Abdominal wall tumor resection + Lysis of Intestinal Adhesions + Retroperitoneal lymphatic tissue dissection. The postoperative pathology was dedifferentiated liposarcoma, and some areas showed rhabdomyosarcoma differentiation and invaded the muscularis of the intestinal wall. Immunohistochemistry: SMA (+), MyoD1(+), Myogenin (+), Desmin (-), Caldesmon (-), S-100(-). The Ki67 positive rate is about 30%. In view of the large range of surgical resection, the patient was transferred to the intensive care unit for transition. The patient was transferred back to the general ward on the 3rd day after surgery and was discharged from the hospital on February 2, 2015.

### 2016-12-08

**Figure 5:**
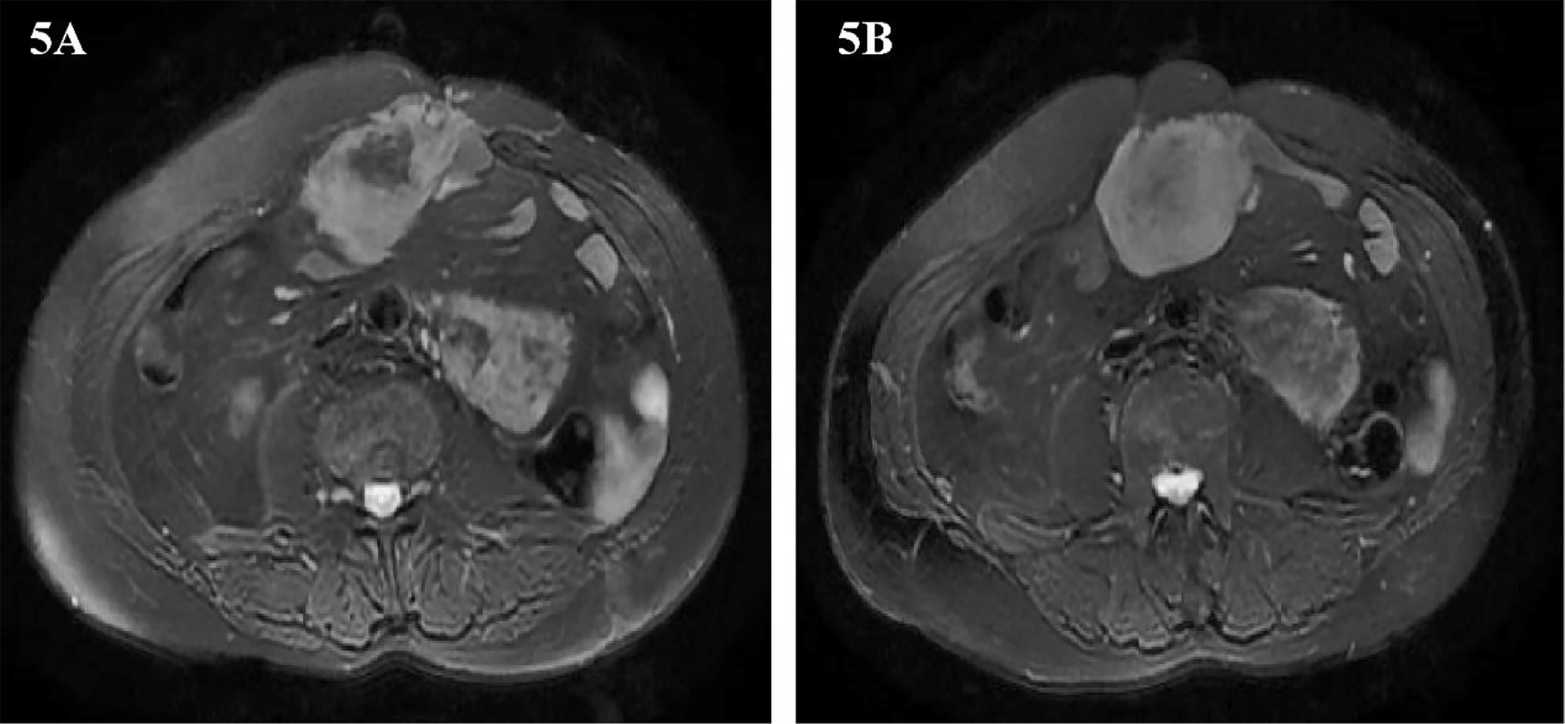
**(A, B)** Abdominal image before the fifth operation.

Two years later, abdominal MRI showed abnormal signal shadow on the lower edge of the spleen again, and metastasis was considered. There were tumors around the operation area and the inner edge of the anterior abdominal wall incision. Multiple space-occupying lesions in the abdominal cavity, considering the possibility of malignant tumor recurrence and metastasis (**Figures 5A, B****)**. The multidisciplinary team of experts (MDT) assessed the patient’s condition and believed that the operation could be performed. Our team performed the fifth operation on the patient on December 8, 2016. Operation name: Retroperitoneal tumor resection + partial splenectomy + partial small bowel resection + intestinal adhesion lysis + abdominal wall tumor resection. The sarcoma encapsulates the distal end of the spleen and occupies a small area. Considering the low risk of the spleen at the margin of resection, it was decided to remove the sarcoma along with part of the spleen. This partial splenectomy is not related to the previous partial splenectomy. The spleen part of the two excisions was small, which did not affect the function of the spleen, and there was no postoperative bleeding. The postoperative pathology was dedifferentiated liposarcoma, some areas showed rhabdomyosarcoma differentiation, and some areas showed malignant fibrous histiocytoma. Immunohistochemistry: Pathological section No. 3: SMA(+), S100(+), CD68(+), MyoD1(-), Myogenin(-), HMB45(-), Ki-67 positive rate is about 20%. Pathological section No. 3: SMA(+), MyoD1(+), Myogenin(+), CD68(-), HMB45(-), S100(-), Ki-67 positive rate is about 20%. The patient was discharged on the 11th day after surgery.

### 2017-06-03

**Figure 6:**
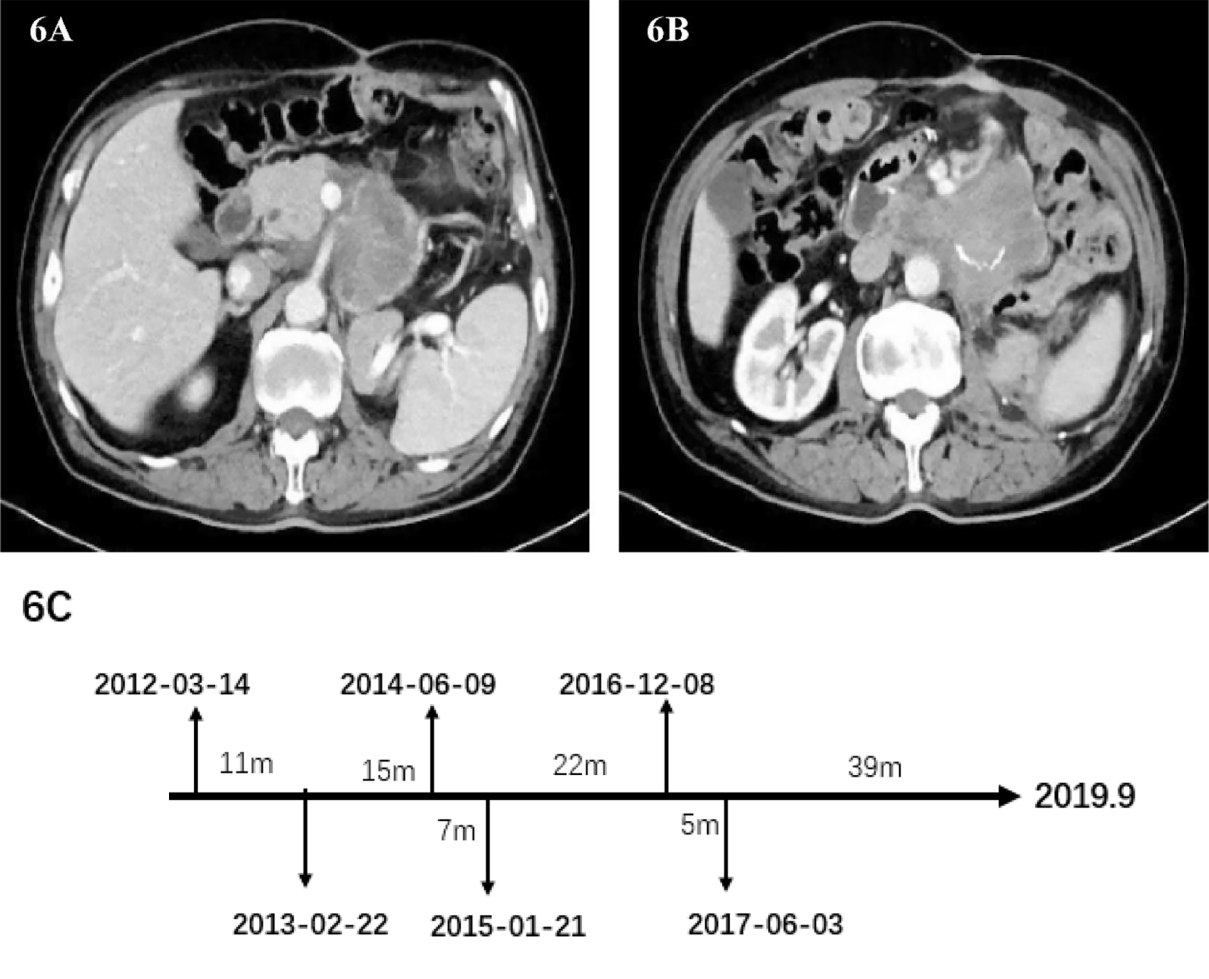
**(A, B)** Abdominal image before the sixth operation. **(C)** Date chart of operation.

In May 2017, the patient’s abdominal CT showed the density of retroperitoneal lump-like soft tissue. Considering the possibility of metastasis or tumor recurrence. There is a space-occupying lesion in the abdominal wall incision, which indicates the possibility of tumor metastasis (**Figures 6A, B**). The surgeon performed the sixth operation which was the final operation on the patient on June 3, 2017. Operation name: Retroperitoneal tumor resection + abdominal wall tumor resection. The history of multiple recurrences and the highly malignant biological characteristics of dedifferentiated liposarcoma made the surgical procedure extremely difficult. The sarcoma wraps the superior mesenteric artery tightly and cannot be peeled off. Considering the risk of continuing surgery, we decided to perform palliative surgery to remove the sarcoma as much as possible. Because the well-differentiated components of the sarcoma are nearly indistinguishable from normal retroperitoneal fat, we surgically removed as much fat as possible in the hope of slowing disease progression. After removing the abdominal wall sarcoma, we performed the incision suture. The abdominal wall incision is closed with a continuous suture using a single-strand absorbable suture. Finally, the incision was reinforced with tension-reducing sutures, and the sutures were removed no earlier than 14 days after surgery. No repair materials or artificial limbs were used in the whole process. Postoperative pathology showed dedifferentiated liposarcoma, some of which showed rhabdomyosarcoma differentiation and invaded subcutaneous tissue. Immunohistochemistry: SMA(-), MyoD1(+), Myogenin(+), CD68(+), S100(-), Ki-67 (+) Approximately 30%. The patient was discharged from the hospital on June 21, 2017. After discharge, he did not receive any radiotherapy or chemotherapy. The patient passed away in September 2019 due to systemic organ failure caused by cachexia (**Figure 6C**).

### Main Points of Vascular Operation

Open surgery should be used when the sarcoma invades the great vessels. For extensive retroperitoneal sarcoma, sarcoma resection should follow an easy-to-hard strategy, from peripheral to central. Since the sarcoma is heavily adhered to the surrounding tissue, you don’t know where to start, but you can separate it while exploring. During the intraoperative isolation of the sarcoma, the blood vessels were accidentally damaged, resulting in persistent bleeding. At this time, the intraoperative visual field was not clearly exposed due to the occlusion of the sarcoma. Bleeding can be stopped with gauze compression. When the visual field is not clear during the operation, electrocoagulation should not be used blindly to cause more damage. Complete hemostasis after the tumor has been completely removed. If the sarcoma has a rich blood supply, the excision process is severely oozing and cannot be continued. A flexible rubber tube can be wrapped around the tumor base and clamped with hemostatic forceps (similar to hepatic hilar vascular occlusion). On the basis of blocking the blood vessels, continue to strip the tissue around the sarcoma, which can reduce the difficulty of the operation. The artery is accompanied by a vascular sheath. Even if the sarcoma invades the abdominal aorta and mesenteric artery, the vascular sheath can be incised to strip the sarcoma, and radical resection can be attempted.

## Discussion

Dedifferentiated liposarcoma has both local invasiveness and the potential for distant metastasis. The 5-year survival rate is 20%-40% (8), and the distant metastasis rate is 15%-20% (9). Compared with well-differentiated liposarcoma, the risk of local recurrence of dedifferentiated liposarcoma is increased by 4 times, and 80% of dedifferentiated liposarcoma will recur within 5 years (10). The histological types of dedifferentiated liposarcoma are diverse, and the appearance of myogenic components may indicate a poor prognosis (11). Surgery is the only way to provide long-term survival benefits for patients with retroperitoneal dedifferentiated liposarcoma (12–14). With the repeated recurrence of sarcoma, the probability of radical resection gradually decreases. The main technical challenges of resection of locally recurring sarcoma are the invasion of multiple organs and the distortion of anatomical relationship (15). Whether the sarcoma can be completely removed depends largely on the degree of invasiveness of the sarcoma and the degree of encapsulation of adjacent internal organs, as well as the surgeon’s accumulated experience in retroperitoneal sarcoma surgery (16). It should be pointed out that due to anatomical distortion and tissue adhesion, the ureter is extremely vulnerable to injury. We usually implant a ureteral stent before surgery to better separate the ureter from the tumor.

### Combined Organ Resection and Vascular Resection

Due to the extensive aggressiveness of retroperitoneal dedifferentiated liposarcoma, combined organ resection is usually required for radical resection. The surgeon needs to make difficult choices during the operation: Should multiple organs and vascular structures be removed? What is the risk of complications after organ removal and blood vessel removal? Will this benefit the patient’s survival? Since the oncological benefits of extended resection may be limited, and the risk of recurrence has not been significantly reduced, a careful surgical plan is required for surgical intervention. When the sarcoma invades the inferior vena cava (IVC) wall in a small range (less than 1/4 of the circumference of the vessel), it can be repaired directly. To ensure that the circumference of the vessel is greater than 1/2 of the previous size, the vessel can be sutured laterally. If the length of the resected IVC is less than 2cm, direct anastomosis can also be performed, but due to the high tension after direct anastomosis, the blood vessels will become oblate, resulting in a certain resistance to the circulation. The lower extremities have edema due to blood circulation disorders, but the symptoms usually recover within 1 to 3 months.

**Figure 7:**
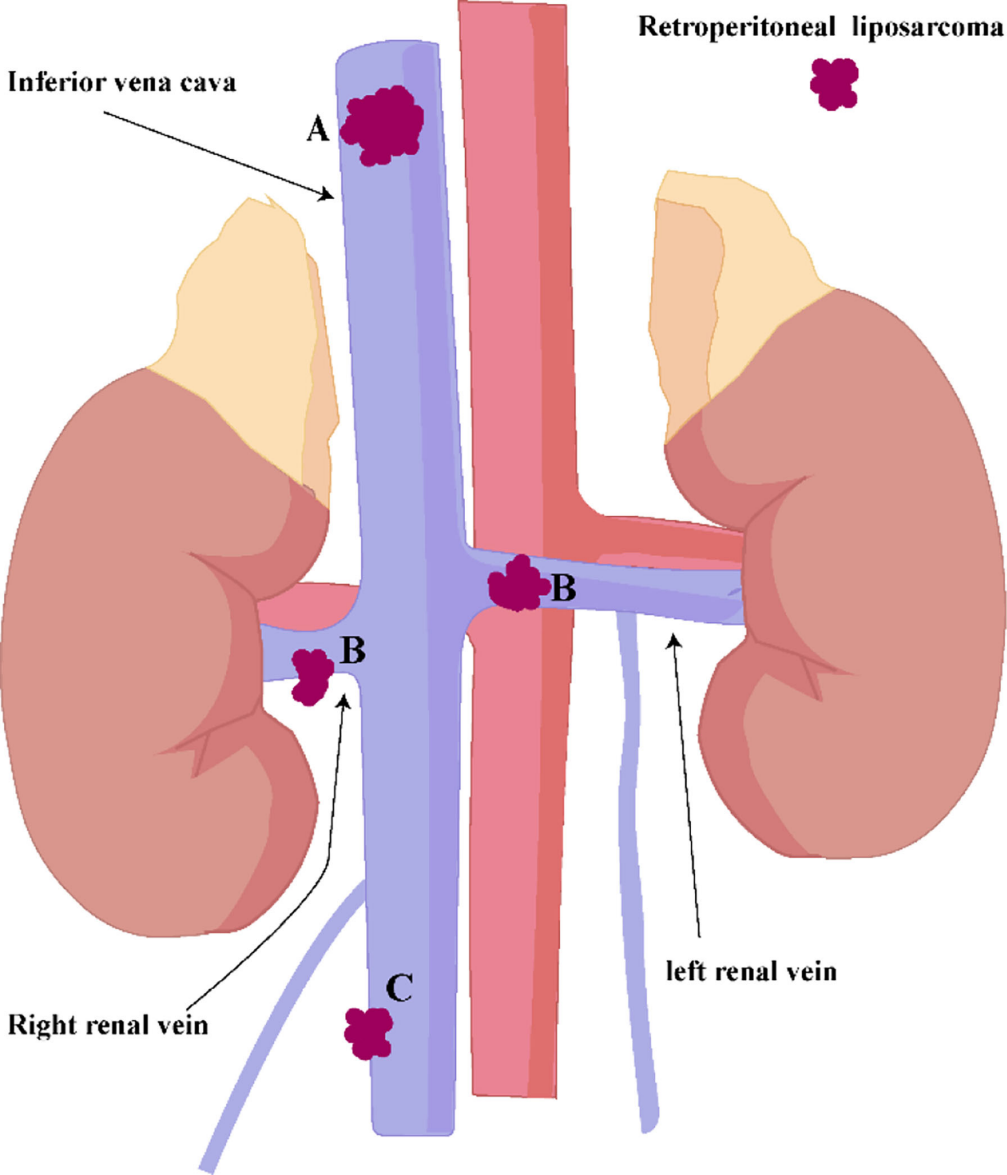
**(A)** IVC above the level of the renal vein. **(B)** IVC at the level of the renal vein. **(C)** IVC below the renal vein level.

The left renal vein has abundant collateral circulation, while the right renal vein has less collateral circulation (**Figure 7B**). Anatomical differences predate that the left and right kidneys are handled differently. Some ideas believed that after ligation of the IVC above the level of the renal vein (**Figure 7A**), the right renal venous return will be blocked due to insufficient right renal collateral circulation. They recommend that the right kidney should be removed concurrently after ligation of the inferior vena cava above the level of the renal vein. We have different opinions. Due to the long-term compression of the superior vena cava by the sarcoma, an effective collateral circulation has been established in the right kidney. In addition, blood from the right renal vein can convect through the inferior vena cava to the left renal vein circulation, so there is no need to remove the right kidney.

If the IVC below the renal vein level (**Figure 7C**) is completely blocked by sarcoma, and there is no obvious lower extremity edema before surgery, the IVC can be resected together with the sarcoma, and it is safe and feasible not to reconstruct the IVC. In this way, the contradiction between anticoagulation treatment after inferior vena cava reconstruction and postoperative wound bleeding can be solved.

### Systemic Therapy

Adjuvant therapy reduces the risk of local recurrence of retroperitoneal liposarcoma, but does not seem to have a significant benefit on overall survival (17–19). Adjuvant therapy does improve survival in some cases. Pathological types, such as synovial sarcoma and rhabdomyosarcoma, are relatively sensitive to standard drugs. However, compared with other histological subtypes of liposarcoma, dedifferentiated liposarcoma is relatively insensitive to chemotherapy (20). Adjuvant therapy will increase the difficulty of operation, make the tissue more prone to bleeding, and it is difficult for the patient to heal the wound after operation, especially the incision requiring intestinal anastomosis, so that the patient will lose the opportunity of the next operation. The patient underwent one cycle of chemotherapy after surgery and developed severe nausea and vomiting. The patient refuses to undergo adjuvant therapy after full communication with the doctor.

After recurrence of indolent Well-differentiated liposarcoma, observation over a period of time before considering resection may be more beneficial to the patient. For relapsed dedifferentiated liposarcoma, systemic therapy is an important intervention. Anlotinib is a novel tyrosine kinase inhibitor that inhibits tumor progression by targeting tumor proliferation, vasculature, and the tumor microenvironment. In the antitumor study of anlotinib against soft tissue sarcoma, the progression-free rate at 12 weeks, median progression-free survival (PFS) and overall survival (OS) in the liposarcoma group were 63%, 5.6 and 13 months. Jianqiang Cai et al. (21) found that patients with liposarcoma appeared to benefit more from anlotinib. Anlotinib is the first multikinase inhibitor to show promising efficacy in liposarcoma. We only started exposure to Anlotinib in 2019, and the patient died in 2019. In addition, immunotherapy has also made considerable progress in the treatment of sarcoma (22), and its efficacy in retroperitoneal sarcoma needs further clinical verification.

### Palliative Surgery

The patient has undergone 6 operations in 5 years, which is not only a test for the patient, but also a great test for our team. Before each operation, our team will struggle with the same question: should we perform another operation for the patient? We believe that the best local control for these patients with recurrent recurrence is surgical resection. If surgery is not performed, the sarcoma will quickly invade the intestine. Eventually, the patient will be unable to eat and endure great pain. Some experts believe that palliative surgery can also improve the long-term survival of patients with advanced retroperitoneal sarcoma. Shibata et al. Analyzed the effect of surgical resection on patients with unresectable retroperitoneal sarcoma and believed that incomplete surgical resection could provide some survival benefits for selected patients (23).

There is evidence (13) that palliative resection may improve survival in patients with symptomatic recurrence. We believe that beneficial palliative surgery should be a failed radical resection, rather than a simple laparotomy that is known to be unresectable before surgery. Palliative surgery that can relieve the patient’s symptoms (such as intestinal obstruction) can indeed show temporary benefits, but this must be carefully weighed against the morbidity after surgical resection. Survival benefit from multiple surgical resections, up to 7-year OS in this patient.

## Conclusion

For complex recurrent retroperitoneal dedifferentiated liposarcoma, surgery should be decided after a thorough evaluation by a professional sarcoma team. The primary site of the sarcoma is an important prognostic factor, with retroperitoneal sarcomas being the most lethal. Due to the particularity and complexity of retroperitoneal sarcoma, a professional sarcoma center should be established to provide individualized treatment for each patient. Radical surgical resection is still the key to the treatment of retroperitoneal dedifferentiated sarcoma. If radical resection of the tumor fails, palliative surgery is also of greater value for patients with symptomatic recurrence. When the patient has symptomatic recurrence, the effect of surgical intervention is even more obvious.

## Data Availability Statement

The original contributions presented in the study are included in the article/supplementary material. Further inquiries can be directed to the corresponding authors.

## Ethics Statement

The studies involving human participants were reviewed and approved by Affiliated Hospital of Qingdao University. The patients/participants provided their written informed consent to participate in this study. Written informed consent was obtained from the individual(s) for the publication of any potentially identifiable images or data included in this article.

## Author Contributions

(I) Conception and design: JG. (II) Administrative support: QX and DH. (III) Provision of study materials or patients: FQ and QL. (IV) Collection and assembly of data: JZ and WF. All authors contributed to the article and approved the submitted version.

## Conflict of Interest

The authors declare that the research was conducted in the absence of any commercial or financial relationships that could be construed as a potential conflict of interest.

## Publisher’s Note

All claims expressed in this article are solely those of the authors and do not necessarily represent those of their affiliated organizations, or those of the publisher, the editors and the reviewers. Any product that may be evaluated in this article, or claim that may be made by its manufacturer, is not guaranteed or endorsed by the publisher.
